# AssociatIion of metabotropic glutamate receptor 5 with amyloid deposition in Alzheimer's disease's patient and mouse models

**DOI:** 10.1002/alz.090779

**Published:** 2025-01-09

**Authors:** Serena Savoldelli, Yanyan Kong, Cinzia Maschio, Uwe Konetiko, Linjing Mu, Jan Klohs, Roger M. Nitsch, Ruiqing Ni

**Affiliations:** ^1^ University of Zurich, Zurich, Zurich Switzerland; ^2^ PET Center, Huashan Hospital, Fudan University, Shanghai, Shanghai China; ^3^ University of Zurich, Zurich Switzerland; ^4^ ETH Zurich, Zurich Switzerland; ^5^ ETH Zurich & University of Zurich, Zurich Switzerland; ^6^ Institute for Regenerative Medicine, University of Zurich, Schlieren Switzerland

## Abstract

**Background:**

Metabotropic glutamate receptor 5 (mGluR5) modulates excitatory glutamatergic synaptic transmission and plays an important role in learning and memory, and in the pathphysiology of Alzheimer’s disease (AD). Here, we aimed to assess the alterations of mGluR5 in the hippocampus of AD patients and mouse model, and the association with amyloid pathology.

**Method:**

Immunofluorescence staining was performed on postmortem brain tissue from 35 AD patients and 36 control patients, as well as on the brain tissue slices from 15 months‐old 3×Tg and arcAβ mouse models of AD amyloidosis. Autoradiography was performed on brain tissue slices from arcAβ mice using mGluR5 tracer [^18^F]PSS232. Proteomic profiling and pathway analysis were performed on hippocampal tissue from six 15 months‐old 3xTg mice and six age‐matched wildtype mice.

**Result:**

Reduced levels of mGluR5 were observed in the hippocampus of AD patients compared to non‐demented control. Ex vivo autoradiography revealed a reduced level of [^18^F]PSS232 in the hippocampus and striatum of arcAβ mice compared to nontransgenic littermate mice. Reduced mGluR5 immunoreactivity was observed near 6E10‐positive Aβ plaques in the hippocampus. In contrast, upregulated levels of Shank3, Grin2a, Grin2b, and Grm5; and upregulation of glutamatergic pathway (GO and KEGG pathway enrichment analyses) were detected in hippocampal tissue from 3xTg mice compared to wildtype mice.

**Conclusion:**

This study revealed a reduction in the level of mGluR5 in the hippocampus of AD patients, and a strain‐dependent alteration of mGluR5 in mouse models of AD.

